# Pantomimic fossils in modern human communication

**DOI:** 10.1098/rstb.2020.0204

**Published:** 2021-05-10

**Authors:** Przemysław Żywiczyński, Sławomir Wacewicz, Casey Lister

**Affiliations:** ^1^ Center for Language Evolution Studies, Nicolaus Copernicus University in Torun, 87-100 Torun, Kujawsko-Pomorskie, Poland; ^2^ Faculty of Science, School of Psychological Science, The University of Western Australia, 35 Stirling Highway, 6009 Perth, WA, Australia

**Keywords:** pantomime, mimesis, semiotics, signed-based communication, evolution of communication

## Abstract

Bodily mimesis, the capacity to use the body representationally, was one of the key innovations that allowed early humans to go beyond the ‘baseline’ of generalized ape communication and cognition. We argue that the original human-specific communication afforded by bodily mimesis was based on signs that involve three entities: an *expression* that represents an *object* (i.e. communicated content) for an *interpreter*. We further propose that the core component of this communication, pantomime, was able to transmit referential information that was not limited to select semantic domains or the ‘here-and-now’, by means of motivated—most importantly iconic—signs. Pressures for expressivity and economy then led to conventionalization of signs and a growth of linguistic characteristics: semiotic systematicity and combinatorial expression. Despite these developments, both naturalistic and experimental data suggest that the system of pantomime did not disappear and is actively used by modern humans. Its contemporary manifestations, or *pantomimic fossils*, emerge when language cannot be used, for instance when people do not share a common language, or in situations where the use of (spoken) language is difficult, impossible or forbidden. Under such circumstances, people bootstrap communication by means of pantomime and, when these circumstances persist, newly emergent pantomimic communication becomes increasingly language-like.

This article is part of the theme issue ‘Reconstructing prehistoric languages’.

## 1. Fossils in the evolution of language

A great many introductions to language evolution begin by highlighting the truism that ‘language does not fossilise’. But this is only truistic in its literal sense, and Derek Bickerton [1]) observes that ‘there may exist contemporary phenomena—living linguistic fossils … that would give us some insight into the processes through which language emerged’. Bickerton's conceptualization of linguistic fossils has since been adopted by language-evolution researchers (e.g. [2,3]) who often highlight syntax as the domain of language where these living fossils can be found, either as specific types of syntactic structures like verb–noun compounds [3] or as structural principles that modern languages still use and elaborate, such as the Agent First, Focus Last type of sentence organization^1^ [2, p 249].

There are some imperfections in this analogy. For instance, palaeontological fossils are specimens and analogizing to them connotes individual linguistic expressions. Living fossils are extant species or other taxa; analogizing to them connotes linguistic clades (e.g. languages or language families). According to Bickerton [1], Jackendoff [2] and Progovac [3], linguistic fossils are more abstract and ontologically seem to be elements of language as such, akin to evolutionarily *conserved organs*. Still, despite its imperfections, this analogy acts as a functional window into the earlier stages of language and is a useful tool for drawing inferences about our evolutionary past.

In the current paper, we propose such a conserved phenomenon, whose history is, however, much longer than that of linguistic fossils. We suggest that the cognitive ability (roughly equivalent to *bodily mimesis*) that was responsible for the emergence of the original sign-based communication in human evolution (§2a) continues in modern humans and is put to work under special conditions. These include situations when people are unable to use language, despite retaining the motivational, cognitive and motor capacities for sharing referential meaning. In line with mimesis theory, we understand this original communication system as pantomime (see §2c), characterized by mimetic communication based on primary iconicity (see §2b) and accordingly refer to its modern manifestations as *pantomimic fossils*. We further argue that if such newly emergent forms of communication continue to be used in interaction, they gradually evolve language-like properties. This also helps explain why the old system did not disappear with the inception of language. Since modern humans are repeatedly confronted with contexts that disable the use of language, pantomime continues as an emergency system for sign-based communication.

In what follows, we first describe bodily mimesis and the nature of bodily mimetic signs, which allows us to describe in detail pantomime as an evolutionary stage in the development of human communication (§2). Next, we discuss pantomimic fossils in modern human communication, referring both to naturalistic and experimental data (§3). Finally, we discuss the observations made in §3, taking a broader evolutionary perspective (§§4 and 5).

## 2. First signs: mimetic and primary-iconic

Many accounts of language origin point to the emergence of symbolic conventions as the most defining event in the evolutionary emergence of language. Although we do not question the fundamental importance of this step, in a recent theoretical proposal [4] we extensively discuss and defend an even earlier breakthrough, one that we term sign-based communication (see §2a below), where signs are understood in accordance with proposals from cognitive semiotics [5–7]. In short, sign-based communication is important because—even before the advent of semiotic conventions—it enabled what many theorists view as some of the key features of language that distinguish it from animal communication: open-ended semantics with displaced reference (in particular [8,9]; also [10,11]).

What would have been the nature of the first sign-based communication systems? For our pre-linguistic ancestors, the first signs to emerge must have been those that could bootstrap communication when no other signs were available (not unlike the ‘symbol grounding problem’ [12]). A compelling theoretical argument can be advanced that the earliest systems of sign-based communication would have relied on *bodily mimesis* as a cognitive mechanism and *primary iconicity* as a semiotic principle (see esp. [4]).

### (a) Bodily mimesis

The increasingly influential theory of *bodily mimesis* ([11,13–15]; see also [5,16,17]) suggests that a generalized cognitive ability of mimesis arose in late australopithecines or early *Homo ca*. 2 Mya. It was originally used for praxis, such as tool production or motor routine rehearsal, then undergoing gradual exaptation for communication. This enabled the use of the body as a representational device, whereby bodily movements could communicate meaning by standing for something other than themselves. Zlatev [18, p 206]) states that bodily mimesis:

… (1) … involves a cross-modal mapping between exteroception (e.g. vision) and proprioception (e.g. kinesthesia); (2) it is under conscious control and is perceived by the subject to be similar to some other action, object or event, (3) the subject intends the act to stand for some action, object or event for an addressee, and for the addressee to recognize this intention; (4) it is not fully conventional and normative, and (5) it does not divide (semi)compositionally into meaningful sub-acts that systematically relate to other similar acts, as in grammar.

Bodily mimesis enables communication that is non-linguistic (points 4 and 5) and uses the body as a communicative device to intentionally transfer referential-propositional information.^2^ An important semiotic consequence of the above definition (encapsulated in point 3) is that bodily mimetic communication must be based on signs. In short, a sign must involve three entities: an *expression* that represents an *object* for a conscious *interpreter* (the three terms are meant in a semiotic sense; for a discussion, see [4]).

Mimesis theory explains sign use in terms of *triadic mimesis*, which emphasizes the role of communicative intentions [11,20]. In triadic mimesis, it is not only necessary that the addressee should understand the meaning of the sign produced by the communicator but they should also realize that the communicator used the sign with the intention (i.e. communicative intention) of facilitating this understanding in the addressee [11,21]. The prototypical examples of triadic mimesis are declarative pointing [10] and iconic gestures [11,20], which, excepting anecdotal evidence, are conspicuously absent in non-human apes in the wild (see e.g. [22] for a review; also §4). In sum, the proposal is that bodily mimesis was the key cognitive precondition for sign-based communication, which is uniquely human and a stepping stone in the evolution of modern human systems of communication, including language^3^. Notably, mimesis theory does not have strong commitments on the specifics of the neural implementation of this cognitive capacity, but it is in principle compatible with Arbib's approach (Mirror Neuron Hypothesis [8], recently updated to Cognitive Neuroprimatology [24]), which stresses the role of the mirror neuron system (see esp. [11]).

### (b) Semiotic considerations

Bodily mimesis implied the use of motivated signs, i.e. signs in which the connection between their expressions and meanings is not pre-established by the shared knowledge of the communicator and addressee but can be inferred from the properties of the expression itself. In this regard, motivated signs differ from conventional signs (or symbols), which are based on the shared knowledge of the communicator and addressee that a particular expression stands for a particular meaning (as for example the lexical label ‘dog’ standing for a particular species of animal) [11].

#### (i) Motivated signs

Motivated signs can be *iconic*, where there is a more or less direct resemblance between the sign's expression and its meaning (e.g. the drawing of a dog brings that animal to mind), or *indexical*, where there is a natural association (e.g. a contiguity-based one) between the sign's expression and its meaning (e.g. the direction of a pointing gesture is associated with the location of a referent) [25]. Typically, all three relations coexist in a single act of sign use but usually one predominates [26]. For example, a pointing gesture typically involves indexicality with respect to its object, resemblance (i.e. iconicity) with respect to the intended gaze alternation or motion of the addressee, and conventionality, since there are different norms for pointing in different cultures [4]. Both iconic and indexical signs allow their users to transmit referential-propositional information, but there is an important difference between the two: indexes are usually tied to situations where the intended referent is available and salient, while icons do not have such constraints ([10]: 233), thus facilitating displacement (i.e. communication beyond the immediate here-and-now [27]). Icons also facilitate open-ended semantics: new signs can be flexibly created to communicate about many semantic domains [8,9].^4^

Mimetic scenarios of the evolution of human-specific communication (e.g. [11,13,29]) acknowledge the role of pointing gestures in this process and link the emergence of pointing to the general characteristic of triadic mimesis, whereby a bodily movement serves to identify an object in the spatio-temporal coordinates of an act of communication [19, pp 363–646]. Mimetic theory, however, puts more emphasis on the role of iconic signs, in particular bodily visual signs, in the evolution of human communication. It underlines the role of imagination, defined as ‘a form of intentionality that is not directed to what is present but to what is absent’, which allows for perceiving the similarity of bodily movements to ‘some other action, object or event’ ([4]; see also [30]). This view on bootstrapping the first sign-based systems is also consistent with approaches that emphasize the importance of gestures in the evolutionary emergence of language (e.g. [8,10,31–33]).

Furthermore, iconicity is not a simple property, but comes in different kinds and degrees [34,35]. For example, signs can be more or less iconic, i.e. their expression can resemble to a greater or lesser degree what they stand for [36]. Hence, at the earliest stage of the emergence of sign-based communication, iconic signs should be maximally similar to the object they stand for (cf. the notion of symbolic distance [37]) so that the connection between the two can be understood without the knowledge of other signs. In semiotic terms, this is *primary iconicity* [36,38], where the similarity between expression and object is instantly recognizable and sufficient for understanding that the former represents the latter. This contrasts with *secondary iconicity*, where this relation is reversed: knowing that a given expression represents a given object is a necessary condition for the similarity to be perceived. Zlatev *et al*. [4] illustrate the difference between primary and secondary iconicity with the example of a sign where a whole body enactment represents the action of hammering (primary iconicity)^5^ and another sign, where the same meaning is represented by a clenched fist of one hand performing the hammering movement against the palm of the other hand (secondary iconicity). In fact, most iconic signs fall along a scale of these two extremes.

### (c) Pantomime

In language origins, mimetic communication based on iconicity is commonly identified with pantomime [8,10,13,39]. Mimetic accounts of the evolution of communication draw attention to the fact that the original human-specific communication was polysemiotic, i.e. consisting of a number of semiotic systems working together [13,29,39,40]. In a recent proposal, Zlatev *et al*. [4] term this system ‘pantomime’ and argue that it included the semiotic systems of gesture, vocalization^6^ and facial expression. Its core component, primarily responsible for the transmission of referential-propositional meaning, was a special form of gesture (broadly understood), which they call ‘pantomimic gesture’. In this paper, which follows up on Zlatev *et al*.'s work, we will refer to ‘pantomimic gesture’ as ‘pantomime’ owing to the fact that in the non-expert sense^7^ as well as in most of the literature on language origins and gesture (e.g. [8,10,43,44]), ‘pantomime’ is understood as bodily visual communication. When we refer to pantomime as a polysemiotic system, we will call it ‘the communicative system of pantomime’.

In the context of language origins, pantomime is characterized by the following general properties (selection based on [40]):

— *bodily visual* modality: movements of the body (usually the whole body) stand for referents;
— *self-sufficiency*: pantomimes are understood without recourse to other signs—typically achieved through *primary iconicity* (as explained above);
— *holism*: pantomimes are typically not analysable into smaller compositional units;
— *improvization*: pantomimes are produced impromptu.

Further, to be a qualitative advancement over communication systems of non-human animals, pantomime must be capable of implementing *open-ended semantics*^8^ with *displacement* (expressing a potentially unlimited range of meanings that are not limited to the here-and-now or to a predefined set of semantic domains).

Primary iconicity and self-sufficiency are crucial. Pantomime should be able to transmit referential-propositional meaning on its own (‘stand-alone’, see [49]). As already noted, self-sufficiency is enabled by robust iconicity, whereby pantomimes should ‘maximally resemble their intentional objects’ [4, p 162]. It should, however, be stressed that the notion of self-sufficiency is to an important extent an idealization. In actual communication, the understanding of even most robustly primary-iconic signs depends on the knowledge of an interactional and wider cultural context ([50], [51]; see also Footnote 5).

To meet the definitional criteria of a sign, the pantomimic expression and object must nevertheless be differentiable by the interpreter. Illustrative in this regard is Gärdenfors's account of demonstration [29,52]. Demonstration is a form of bodily mimetic communication in which a teacher performs an action for the benefit of a student. It is defined as follows:

— (D1) The demonstrator actually performs the actions involved in the task.
— (D2) The demonstrator makes sure that the learner attends to the series of actions.
— (D3) The demonstrator intends that the learner perceives the right actions in the correct sequence.
— (D4) The demonstrator exaggerates and slows down some of the actions in order to facilitate the learner's perception of their important features. [29]

If we substitute ‘communicator’ for ‘demonstrator’ and ‘addressee’ for ‘learner’, the only essential difference between demonstration and pantomime is feature (D1): in demonstration the teacher actually performs the actions involved in the task, while in pantomime the communicator pretends to perform the actions, which makes pantomime a form of pretense. Since demonstration, e.g. of hammering, is hardly distinguishable from the praxic action of hammering, it cannot be a sign. By contrast, a pantomime of hammering is clearly distinguishable from the praxic action it stands for, and further, there is an asymmetric relation between the two [5,36]: the pantomime stands for the praxic action but not vice versa.

There have been attempts to describe in more detail the type of iconic bodily visual signs (iconic gestures in the broad sense of the term) that meets the criteria set for pantomime in language-origins literature [8,10,29,40]. Zlatev *et al*. [4] used distinctions present in the literature on gesture (e.g. [43,53–56]) to define the characteristics of pantomime, including the properties of primary iconicity and self-sufficiency:

(a) a dominant use of primary iconicity [57], where the similarity between the gesture and what it represents is largely sufficient for establishing the reference;
(b) a dominant use of whole-body movements, rather than hands-only movements [40];
(c) a dominant use of the first-person perspective, which consists in the explicit or implicit mapping of the whole body onto the represented object (even if only a part of the body is foregrounded [58])^9^;
(d) a dominant use of the enacting mode of representation, with the body of the gesturer mapping onto the (human) body of the referent [56];
(e) a dominant use of gestures standing for objects and actions in peripersonal space, i.e. the space immediately surrounding one's own body [59].

Zlatev *et al*. [4] consider all of these properties as clines, which indicate the extent to which an individual gesture can be described as pantomimic, and argue that in actual communication, pantomimes do not have to exhibit all of them. For example, since many everyday actions (e.g. walking, pushing, jumping) involve coordinated muscular activity across the entire body, to represent these as iconically as possible would require a similar use of the whole body [40]. But actions performed by specific body parts, e.g. eating, will be pantomimed by movements of these body parts, rather than movements of the whole body.

Zlatev *et al*. [4] use the above characterization to speculate about the evolution of pantomime, which consisted in increasing the role of (a’) secondary iconicity, (b’) hands-only movements, (c’) third-person perspective (see Footnote 9), (d’) the tracing and embodying modes of representation^10^ and (e’) gestures standing for objects and actions in extrapersonal space, i.e. the space far from one's body. It ushered in a gradual transition from the communicative system of pantomime (including vocalizations) to post-mimetic communication, one of the latest manifestations of which was language. This transition occurred through ‘a long biocultural spiral of conventionalization’ [11], but it was cultural evolution that played the dominant role in this process (cf. Zlatev's [11] notion of a ‘post-biological phase of evolution’). Once sign-based communication had been established, pressures for expressivity and economy first led to a simplification of the form of signs and a stabilization of their connection to specific meanings (post-mimesis 1). These processes allowed for the expansion of semantic space and the emergence of semiotic systematicity or combinatorial expression (post-mimesis 2), which is a more efficient way of coding meanings in a large meaning space [11,60]. These changes entailed a gradual reduction of iconicity (transition from primary to secondary iconicity)^11^, which tends to hinder combinatorial expression [62] and is less suitable for expressing general or abstract meanings [63].

## 3. Pantomimic fossils

As explained in §1, we take inspiration from the ‘linguistic fossils’ approach and extend it to communicative phenomena more broadly. We argue that the capacity for bodily mimesis persists in modern humans, and so we expect to find pantomime in circumstances where, for a variety of reasons, people are deprived of the ability to use (spoken) language. Below we discuss naturalistic and experimental evidence from five such types of situations. Our review is guided by the questions relating to how these emergent forms of pantomimic communication, ‘pantomimic fossils’, differ depending on specific bootstrapping contexts and what they tell us about the original bootstrapping situation in our phylogeny.

### (a) Travelogues

An interesting line of evidence comes from the travelogues of European discoverers during the Age of Exploration (15th–17th centuries), who recorded their contacts with indigenous peoples. Importantly, such encounters were brief, which prevented interactants from learning even the rudiments of each other's languages or more broadly, culture. Hence, these encounters can be taken as examples of the situation when people in the absence of shared conventions have to improvise communication. A rich source of such data is the chronicle *The Principall Navigations, Voiages, Traffiques and Discoveries of the English Nation* compiled by Richard Hakluyt (1553–1616), which contains reports of 15th- and 16th-century English travellers and is considered one of the most important texts documenting the Age of Exploration [64].

While the authors do not discuss the details of how they communicated with members of the indigenous populations, their descriptions indicate that ‘gesture’^12^, sometimes whole-body gesture, constituted the primary means of communication, which was supported by facial expressions and vocalizations (both non-linguistic and linguistic, the latter however not being understandable to the local population). Consider the following passage in which the English party is greeted by a chieftain of the tribe they visited: ‘… he beckoned us to come and sit by him, which we performed: and being sat hee made all signes of ioy and welcome, striking on his head and his breast and afterwardes on ours, to shew wee were all one, smiling and making shewe the best he could of all love, and familiaritie’ [65, p 331].

One of the most common themes in the travelogues is communicating one's intentions upon first contact and plans for future actions, as in: ‘For which losse they yet sorrowed, shewing with signes, that one day they would be revenged: that done, we came to our ships againe’ [65, p 144]. However, the breadth of topics communicated in this way extends well beyond this domain. Topics frequently raised after contact was established involved trade arrangements concerning the type of goods to be bartered, their quality and price. More surprisingly, the parties were able to communicate even about relatively complex subjects, related to *technology, topography, political affairs* or *religion*. Here, the parties discuss foodstuffs—how they are grown and how to prepare them for eating: ‘Of those things they have, they would with signes shew us how to dresse them, and how they grow’ [65, p 107]. The travellers often inquired about the dangers that awaited them and were given instructions, sometimes very detailed, on how to avoid them; for example: ‘Moreover they shewed us with signes, that the said three fals being past, a man might sayle the space of three monethes more alongst that River …’ [65, p 141–142] The visitors also managed to gather information about natural resources of the lands they were visiting: ‘They shewed unto us by signes that they had in the lande golde and silver and copper, whereof wee have broughte some home’ [65, p 209], or about local politics: ‘… they gat it by force of armes of the inhabitants of the place, named by them Thimogoa, their most ancient and naturall enemies … ’ [65, p 527]. Most strikingly, the travellers were able to understand the basic philosophical concepts of indigenous people, as in this report: ‘They have no knowledge of God, nor of any religion, saving of that which they see, as the Sunne and the Moone’ [65, p 485].

These travelogues provide a rare, first-hand record of the bootstrapping process taking place in a natural setting, where both parties communicate about their vital needs in the absence of any knowledge of each other's language or culture. Though unsystematic, these accounts provide evidence that modern humans bootstrap communication primarily relying on improvised gestures, with occasional mentions that they were whole-body gestures. There is no information that could help identify which specific types of gestures were used, but we can speculate that at least some of the topics (e.g. topography, manners of preparing food, etc.) necessitated the use of sequences of iconic signs. The breadth of topics also shows that such visual-bodily, improvised communication was semantically open and capable of expressing displaced reference (e.g. plans for the future or the topography of distant locations).

### (b) Charades

Another naturalistic situation where the absence of language leads to spontaneous emergence of pantomimic communication involves games in which language use is temporarily blocked. In these games, players are required to communicate meanings to teammates, but are prohibited from using language, which forces them to rely on non-verbal means of communicating, and typically pantomime. For example, the popular party game of *charades* requires one person (the ‘actor’) to act out a word or phrase without relying on speech or writing, while the other players (the ‘guessers’) attempt to guess that word or phrase (e.g. [66], see also [67]).

The popularity of such games testifies to the considerable expressive power and versatility of forms of pantomimic communication. For example, Hidayati [68] showed charades to be an effective method for teaching vocabulary in English as a Foreign Language class. Jeffreys [69] describes an application in doctor–patient communication in which role-playing charades were used to teach the utility of a range of non-verbal means of conveying medical conditions and emotional states. Peacock *et al*. [70] report a version of charades to be effective at instruction about the utility of different reward systems in the context of management and organizational behaviour. Pavlov & Yatsenko [67] describe what they call ‘The Babel Experiment’, a method of using pantomime to achieve a more in-depth understanding of underlying concepts in an abstract topic: software development.

We note that this communicative effectiveness is not accomplished completely independently of language and conventional signs more generally. The essence of charades and similar games consists in prohibiting the use of language for ‘online’ communication, thereby offloading referential communication to gesture, both iconic and indexical, which must be self-sufficient for the guessers to identify the intended meaning. Interestingly, the actor in charades typically adopts a standing position, which facilitates the performance of a whole-body pantomime [68]. The use of this system of communication immediately instigates the pressure for expressivity, which leads to a rapid evolution of that system. Notably, conventional signs, such as emblematic representations of the numbers of words in the target phrase, often arise spontaneously in such games [40], in parallel to similar situations in experimental settings (see §3e below).

Again, there are important limitations in using charades to illustrate the original pantomimic communication and its further development. Like the communication developed by people with aphasia (but for different reasons; §3c below), in charades, the non-linguistic transfer is scaffolded by a rich linguistic and lingua-cultural context. Participants typically draw on bodies of linguistically coded cultural knowledge, and the setting of the game, the instructions, the items to be guessed and the guesses themselves all have linguaform. This is best illustrated in the use of puns (such as ‘metaphysician’ reanalysed and shown as ‘met a physician’, etc.—[71, p 271]). Finally, the use of other conventional signs—in particular emblematic gestures—varies but is typically allowed in such contexts to a significant extent.

### (c) Language impairment

The original human system of mimetic communication based on pantomime^13^ may also become useful in cases where, owing to a variety of medical conditions, people have experienced a partial or total loss of language (for a review, see [13, pp. 198–200]). For example, there is evidence that people with moderate and severe aphasia can overcome their language deficits by spontaneously building a substitute communication system using pantomime, pointing, facial expressions [76,77] and other iconic forms of expression, such as drawing [78]. Even in cases of severe language loss, people retain the ability to use pantomime both receptively and productively [79] and are able to engage in pantomimic communication naturally in interpersonal contexts [80,81]. There is an ongoing debate in aphasiology about how specific lesions affect pantomimic capabilities, which leads to a more general question of the separability of the cortical infrastructure for language and gesture (cf. [13, p 198], [43, pp. 332–343], [82]). For example, some research shows that lesions to left frontal regions (associated with Broca's aphasia) adversely affect the ability to pantomime tool use, whereas lesions to left parietal regions (associated with Wernicke's aphasia) leave this ability intact [83].

The resilience of pantomime in situations of language loss [84] has important therapeutic implications, as pantomime (typically used in combination with the other semiotic systems mentioned above) is often used as a platform for successfully rehabilitating patients afflicted by aphasia or similar conditions. One of the most successful rehabilitation methods is Total Communication Training, which operates on the ‘catch-as-catch-can’ principle: patients are invited to use any communicative means available to them, including sparse words or wordlists, but rely mostly on non-verbal visual communication—pantomime, drawing, photographs and other material props [81].

Neuropathological research, and particularly the study of aphasia, can shed light on the problem of bootstrapping communication. The bulk of the neuropathological evidence suggests that in situations of language loss people heavily rely on pantomime. Such communication emerges spontaneously, although it can be encouraged for therapeutic reasons and, at least to some extent, is able to substitute language. For example, people can use this form of communication to describe both concrete and abstract concepts, past events or plans for the future (open-ended semantics with displacement) [85]. It also corroborates the view articulated in mimesis theory that mimetic communication arose prior to and is in principle independent from, post-mimetic communication, including language [14]. This view is further supported by research on apraxia and particularly ideomotor apraxia, which specifically targets the production of pantomime [86]. These lines of evidence give us grounds to argue that once cerebral damage leads to language loss, pantomime is used to facilitate communication.

However, data from neuropathology should be approached with caution. Apart from acute cases of global aphasia, language impairments rarely result in a complete loss of linguistic abilities [87]. People usually retain some ability to produce or comprehend language, or both, to some degree. Further, people with aphasia live in a linguistically rich environment, mainly interacting with individuals whose linguistic abilities are unimpaired. One such context is therapy, which very often makes use of language-based techniques (e.g. [88,89]). Hence, language loss owing to cerebral damage does not correspond to the original bootstrapping situation in phylogeny, both with regard to the users' communicative abilities and the social context in which communication takes place.

### (d) Emerging signed languages

One of the most important insights into the processes of the emergence of linguistic communication comes from special populations for whom it is not possible to use a shared spoken language. Particularly, newly emerging signed languages provide researchers with a unique opportunity to see language being created *de novo* in real time and in a natural setting [90]. Such languages are usually divided into the deaf community signed languages and village signed languages. The former, e.g. Nicaraguan Sign Language (NSL), Isreali Sign Language (ISL) or Sao Tome and Principe Sign Language (STPSL), emerge when deaf people from different geographical locations are brought together for educational reasons [91,92]. Village signed languages, e.g. Al-Sayyid Bedouin Sign Language (ABSL), Kata Kolok, Adamorobe Sign Language and Alipur Sign Language, develop in small, isolated communities, in which there is a high incidence of deafness [91,92].

These two types of conditions show how signs are initially bootstrapped. Whereas the first signs in village signed languages develop communally from interactions of communicating individuals, for users of a deaf community signed language, the bootstrapping process would normally start with ‘home signs’, which they developed before entering a school or an educational facility [92,93]. Such ‘home signs’ are spontaneously created gestures to facilitate communication between deaf children and their hearing family members [94,95]. They are based on indexical and iconic gestures [96], are not limited to specific semantic domains and are capable of displacement (e.g. used to build narratives; [97]). Initially, they are robustly iconic [98] and to some degree improvised, in that they lack systematic pairing between expressions and intended meanings [99] and are concatenated into strings [94,100]. Later, the expression–meaning relations stabilize, which opens the door to conventionalization [101], and elements of language-like systematicity develop, such as consistent word order or rudimentary morphology [102]. When home-signing children are brought together, the lexicon gradually acquires inter-user consistency, usually with one of the competing home signs being selected [92].

The general mode of sign creation (in both semiotic and signed-linguistic senses) is similar in deaf community signed languages and village signed languages. Signs at the early stage^14^ are motivated and most of them are robustly iconic. For example, signs for actions often make use of pantomimic enactment (e.g. the sign for ‘strike’ in early ABSL, [103]; or the sign for ‘eat’ in STPSL, [92]), which can be interpreted as self-sufficient and primary-iconic signs. Such signs commonly involve the first-person perspective and peripersonal space (see §2c). There are, however, other signs that do not conform to this characterization (e.g. the sign for ‘hit’ in early ABSL; [103]), such as those that make use of other modes of representation than enactment (usually, tracing and representing), involve the third-person perspective and extrapersonal space; hence, their interpretation as primary-iconic is problematic. Further, early signs typically have a large signing space and engage actions of both hands, the head and trunk [92,105,106]. Signs are holistic in the sense that the expressive movement as a whole stands for the intended meaning [92,93,103]. The only complex morphological units seem to be compounds, which consist of combinations of holistic signs (e.g. in STPSL: BANANA + TREE = BANANA TREE, WOMAN + CHILD = GIRL; [92], [107]). Like home sign communication, utterances consist of either one sign [108] or concatenations of signs [92], but exhibit a predominant word order [92,108,109].

Subsequent evolution of signed languages sees the reduction of pantomimic elements. A good illustration of this process is the evolution of the sign AIRPLANE in STPSL [92]. Initially, the sign was produced with open arms standing for the wings of the airplane and the rest of the body for the fuselage, and hence exemplified personification, a special type of pantomimic enactment^15^. Instead of enactment, the new sign (a short movement of the dominant hand in front of the forehead) employs the embodying strategy (the hand stands for the airplane as a whole—[56]) and third-person perspective. The original whole-body form has been reduced to manual expression, which has also resulted in the reduction of the signing space. These processes lead to a gradual conventionalization of signs, whereby the relation between the sign's expression and meaning depends more and more on shared knowledge than the characteristics of the expression itself [90,92]. The growing economy in sign production makes it possible to develop functional differentiation between the articulators so as to convey increasingly complex linguistic functions [105]. While the hands and arms become primarily responsible for the transmission of lexical meanings, actions of the face, head and trunk can be used simultaneously to code grammatical information such as tense, mood, modality, shared reference or thematic structure ([104,108,110]; cf. the notion of ‘dedicated gesture’ [103]).

These observations do not do full justice to the complexity of the processes involved in the emergence of signed languages. Here, we have mainly targeted these elements that document the creation of early signs to see if they have the characteristics of pantomime^16^ (see §3). Indeed, they are typically holistic (with no morphological structure), robustly iconic and their production involves both hands and other body parts (mainly the trunk, head and face, which do not, however, have any grammatical function), which is often related to the large signing spaces that early signs make use of. As a semiotic system, a signed language at the early stage of development is capable of open semantics and displacement. Regarding the properties of primary iconicity and self-sufficiency, the pantomimic status of early signs is more a matter of the cline described by Zlatev *et al*. [4]. Some of the examples that we discussed can be interpreted as primary-iconic; others, although highly iconic, do not seem to show enough iconicity to be interpretable without a specific communicative context.

While we assume that pantomime is responsible for the early, bootstrapping phase in the development of sign-based communication, the evidence from emerging signed languages often lacks information of such circumstances. Sometimes, as in the case of village signed languages including ABSL^17^, there is little data on what the very first signs looked like. More importantly, users of emerging signed languages are socialized in linguistically, or more generally semiotically, rich environments. Both home signers and users of village signed languages engage in communicative interactions with users of spoken languages and are familiar with the cultural conventions of their communities; hence, the need for self-sufficient and primarily iconic signs even at the bootstrapping stage of the emergence of signed languages may not be as pressing as it was in our evolutionary past.

Beyond the bootstrapping phase, the development of emerging sign languages also seems to follow the general trajectory predicted by pantomimic scenarios. A gradual conventionalization of signs is accompanied by a gradual decrease in their iconicity. Signs become more economic, i.e. they require a smaller signing space and are mainly produced by the hands and arms, which allows other articulators, mainly the trunk, head and face, to be gradually recruited for expressing grammatical information. At a higher level of granularity, some evidence from STPSL, which is at a very early stage of its evolution, may suggest a tendency for the enacting strategy and first-person perspective to be replaced by other representational strategies and the third-person perspective. However, we note that there is not enough empirical ground to argue that such changes generalize to the dynamics of signed language emergence.

### (e) Experimental research on communication

Naturalistic data show that modern humans can revert to motivated signs, including pantomime, when they are unable to use spoken language (e.g. [94,95,104,111,112]). Recent experimental studies offer a way to corroborate these naturalistic observations. These studies often take the form of *experimental semiotics*, a paradigm in which participants are prohibited from using language and are asked instead to create communicative conventions from scratch [113]. The popularity of experimental semiotics has grown rapidly in recent years (e.g. [114–118]), as they enable researchers to approximate the circumstances that our pre-linguistic ancestors faced when they attempted to communicate without a preexisting language. By stripping modern humans of their verbal communication and asking them to invent novel signs, researchers can observe new communication systems emerging and evolving in real time through interaction under controlled conditions.

In the classic design of such studies, participants engage in a ‘guessing game’, during which they have to use a pre-selected semiotic resource (e.g. drawing [119]) to improvise signs standing for objects taken from a closed inventory (e.g. a microwave from the inventory of different household devices). Usually, one participant is asked to provide a sign, and the other to guess which object from the inventory has been represented (for a review of the methodology, see [120]). Collectively, these studies show that initially signs are highly iconic, rich in detail and idiosyncratic. In the course of interactions, signs become simpler and conventionalized, i.e. the same form is used by the interactants (for a review, see [121]).

The decisive factor in the emergence of conventional signs is repeated and interactive use. For example, Garrod *et al*. [119] had pairs of participants graphically communicate a series of easily confusable items (e.g. a microwave, television or computer monitor), and either allowed them to interact with each other or not. With even a very minimal level of graphical interaction (e.g. the use of a tick to indicate comprehension), partners' drawings converged and developed from primary-iconic and idiosyncratic signs to simpler and more conventionalised signs—this tendency being more pronounced when players alternated roles in drawing and guessing. In the non-interactive condition, drawings remained iconic and, instead of becoming simpler, their complexity increased. The authors argued that interaction promotes a shift in the locus of information from the properties of the expression (e.g. the degree of its similarity to the intended object) to the users’ memory, allowing the signs to become simpler [122].

The results obtained by Garrod *et al*. [119] and in similar studies in drawing-based referential communication tasks (e.g. [123–127]) provide evidence that the initial stage of the bootstrapping process depends heavily on iconic signs, and that repeated, interactive use of iconic signs promotes their conventionalization and simplification. Paring back signs to make them simpler often leads to a reduction in overall iconicity, as non-essential elements of the sign are stripped away ([119,128]; see also [50]). Natural language tasks reveal a similar pattern: participants asked to describe abstract shapes to a partner initially tend to use detailed figural descriptions; over time, these descriptions are refined, becoming briefer and more abstract [129]. Supporting these findings, other experimental studies show that communication suffers when the production of iconic signs is restricted or prohibited (e.g. [130–132]).

An important line of research that grew out of the model of repeated interactions investigates which semiotic systems work best when language is prohibited. For instance, Fay *et al*. [115,133] had a cohort of adult participants try to communicate lists of words to a partner without using language or conventional signs. Participants used either bodily visual communication (movements of the hands, body and face) or vocal communication (non-linguistic: sounds that are not words). Participants guessed more of their partner's signs correctly when their partner used visual-bodily signs compared to vocal ones; interestingly, combining visual and vocal signs did not lead to more successful communication than using only unisemiotic, gestural signs (cf. [39]; for a review, see [134]; but see [135]). These findings have since been replicated in children [136,137].

Another line of experimental research investigates the iconic potential of vocalization. A number of studies [138–142] using the ‘guessing game’ paradigm (see above) or the ‘foreign language’ paradigm [143] have shown that vocalizations have the capacity to be iconic. Hence, the authors argue that the vocal system might have played a role in protolanguage (for a review, see [144]). However, the bulk of studies investigating the problem of semiotic systems in emerging communication supports the findings made by Fay *et al*. ([117,133]; see above) about the superiority of iconic gesture over vocalization in getting communication off the ground (for a review, see [134]). The model of sign creation developed by Fay together with Lister [128] seeks to explain these results by highlighting the iconic potential of bodily visual communication, whereby highly iconic visual signs (cf. primary iconicity) are able to transmit referential information even in the absence of any shared communicative conventions (cf. self-sufficiency). Once a mutual understanding has been achieved, the pressure to communicate through iconic signs diminishes. Instead, there is a drive to refine the sign system, making it more efficient [128].

With regard to the problem of pantomimic fossils, experimental research provides two valuable insights. First, modern humans are able to communicate by means of improvised bodily visual signs with relative ease, and second, repeated communication through the use of such signs promotes their simplification (leading to a gradual decrease of their iconic character) and conventionalization. At the initial stage, signs meet all the major characteristics of pantomime: they are improvised and holistic bodily visual forms of communication capable of expressing meanings that are not limited to selected semantic domains and the here-and-now.

However, some of these characteristics should be interpreted with caution, mainly owing to the limitations of experimental designs. Typically, experiments use a closed meaning space, and as a result, they do not show, at least directly, that such communication has open semantics. This limitation also bears on the status of emerging signs as primary-iconic and self-sufficient, i.e. their having the ability to perform the referential function only by virtue of their similarity to intended referents. The complexity of semantic information is likewise constrained. Apart from a few studies in which participants were asked to communicate events (e.g. [39,145,146]), experiments are usually focused on the communication of individual objects corresponding to lexical labels. For example, Nölle *et al*. [147] point to the constraint of many experimental-semiotic studies in that they do not allow participants to use deictic signs. Even experimental logistics may impact the characteristics of emerging signs. Most experimental studies employ a paradigm called ‘silent gesture’, alternatively referred as ‘elicited pantomime’ (e.g. [148–150]), where participants are encouraged to use primarily their hands, either by express instruction [46] or by being made to take a seated position, which constrains bodily movements (e.g. [115,116]). However, in studies where participants were allowed to take a standing position, they usually produced whole-body pantomimes; interestingly, they then resorted more often to the enacting strategy than to representational or tracing strategies, and adopted the first-person perspective more often than the third-person perspective [39].

## 4. Discussion

To evaluate the claim of the role of pantomime in bootstrapping communication, we have looked at different situations in which the use of language is impossible or severely limited. The evidence presented above indicates that when facing such restrictions, modern humans are able to bootstrap communication naturally and without prior instruction. Emerging communication systems are based on improvised signs and are semantically advanced, in that they can transfer referential-propositional information not restricted to either specific semantic domains or to the here-and-now. Further, we saw that the most effective bootstrapping strategies (§3e) rely on bodily visual signs that are holistic and highly iconic. Together, these findings suggest that communication that emerges at the bootstrapping stage when language is blocked has all the major characteristics of pantomime (see §2c), but only if a number of caveats are addressed.

Most importantly, pantomimic scenarios derived from mimesis theory (but see also [10]) emphasize that first signs should exhibit high degrees of primary iconicity, allowing them to transfer information in the absence of any communicative conventions (self-sufficiency). The results of many experimental studies corroborate this view, but, as we noted, such an interpretation of them is mitigated by the fact that emerging signs are only self-sufficient to the degree that they are distinguishable from other items in a closed meaning space provided by the experimenters. There is also evidence that primary iconicity plays an important role in bootstrapping early signs in emerging signed languages. Although there are many examples of early signs whose status as primary-iconic is problematic, we would like to draw attention to the fact that pantomime is expected in the very first signs that appear in the process of bootstrapping. In village signed languages, such as ABSL, even the earliest recorded signs may already have undergone some conventionalization, whereas in the deaf community signed languages, such NSL or STPSL, initial conventionalization would have occurred at the stage of home-signing, which could have an impact on how signs were bootstrapped after home signers arrived at school. Finally, the self-sufficient and primary-iconic nature of signs in charade games or the pantomimes of people with aphasia can be questioned on the grounds that these forms of communication still rely on language in one way or another.

All these reservations illustrate a more general point. As modern humans, we are in a very different situation from that of our ancestors using polysemiotic pantomime. Probably the closest approximation is described in the travelogues (§3a), which unfortunately are sparse in detail. Particular contexts in which language is blocked set different bootstrapping requirements, and we should expect that the use of pantomime will be calibrated to these requirements, both with regard to its fundamental and more specific characteristics (e.g. whole-bodiness or enacting mode of representation). Together, the evidence discussed in §3 suggests that pantomime works better than any other semiotic system at the very first stage of bootstrapping sign-based communication.

A closely related and equally important point is that pantomime is also very transient: it is uneconomical in terms of production costs and error-prone in terms of comprehension, and thus under strong pressures for conventionalization. In particular, the evidence from emerging signed languages and semiotic experiments (§§3d and 3e) supports the view that pantomime is a good substrate for rapid evolution of linguistic features, such as complex lexicon, morphology and syntax (see esp. [151]). This view is consistent with approaches that underline the decisive role of cultural evolution in the emergence of language (e.g. [10,42,152–154]). In particular, our understanding of both the evolution of pantomime in human phylogeny and the evolution of pantomimic communication in modern humans can benefit from iterated learning studies, which focus on the role of learning biases in shaping communication systems (for a review, see [152]). They identify two major pressures responsible for the emergence of linguistic properties like double articulation (phonology) and compositionality through cultural evolution: ‘a pressure for simplicity arising from a domain-independent bias for compressibility in learning, and pressure for expressivity arising from language use in communication’ [155]. Iterated learning experiments show that the combination of the two pressures—the first simulated by vertical transmission between generations of learners; the other, by interactions between learners from the same generation—leads to the emergence of language-like structure [155,156].

This account, implying the evolution of language from pantomime, mostly through cultural evolution, leaves several important questions unaddressed. First, globally the most prominent manifestation of language is speech. Accounting for this fact is notoriously difficult for all gestural and pantomimic approaches to language origins. This difficulty, sometimes referred to as the ‘modality transition problem’, has long been recognized by language-origins theorists (e.g. [157–159]; see esp. [160], pp. 433–465), and we extensively discuss it in our previous work (esp. [161], pp. 235–276). While no single convincing counter-argument has been elaborated to solve the ‘modality transition problem’, it is crucial to note that mimetic-pantomimic theories are much less vulnerable to this problem than gesture-first theories (Wacewicz and Żywiczyński in press [49]), in that they do not posit a transition from gesture to vocalization but rather from an original system of communication to a current one, both of which are essentially polysemiotic. As we have explained in §2c, the original communicative system of pantomime included not just bodily visual signs (although these were the core element responsible for the transmission of referential-propositional meaning) but also other semiotic systems: vocalization, facial expression, and possibly the rudiments of depiction [4]. The transition from pantomime to modern communication consisted in a gradual reconfiguration in the division of labour between these semiotic systems, with vocalization becoming primarily responsible for the transmission of referential-propositional information [38].

Another question is about the evolutionary trajectory/continuity from the ‘LCA-c baseline’ (the cognitive-communicative abilities we assume as already present in the last common ancestor of humans and chimpanzees) to full human languages or at least to a hypothesized protolanguage [2]. Like triadic mimesis, pantomime seems to be *almost* present in non-human apes, or in other words, to fall in what Donald [14] and then Zlatev (e.g. [18]) call their ‘zone of proximal evolution’. On the one hand, they have the necessary prerequisites for pantomime (in particular, flexible motor control over one's own body and rich intentional bodily communication; for a review, see [162]), but on the other hand, in apes ‘…pantomiming is conspicuously absent, apart from isolated anecdotes' ([163], p 136)^18^. We suggest that the best explanation for the lack of pantomime in apes may start not with proximate-level reasons (e.g. representational or cerebral implementation) but rather ultimate-level ones (related to evolutionary fitness); and we develop this specific argument in other works (esp. [166]), following other influential accounts [10]. In short, apes do not live in a social ecology that would make cooperative communication evolutionarily stable, and as a result, they likely lack the motivational factors for engaging not only in sign-based communication (e.g. [167]) but in fact in any form of large-scale cooperative communication (e.g. [168]).

## 5. Conclusion

One of the most important insights coming from the theory of evolution is that we carry our evolutionary history within ourselves. This implies both continuity and changeability, encapsulated in the Darwinian principle of inheritance with a variation. The science of language evolution accepts this logic: in important ways, the communicative and cognitive systems of our ancestors both resemble modern human communication and cognition and differ from them. In line with mimesis theory [4,13,29], we suggested that modern humans' language-based communication evolved from pantomime, understood as a polysemiotic communicative system dominated by (primary) iconicity. The most important continuity between pantomime and language is that both are based on signs, implying conscious differentiations between expressions and meaning by communicators [4].

The discontinuity concerns the properties of signs: the original system of pantomime depended on motivated, and most importantly iconic, signs, while language primarily uses conventional signs, which can be combined in complex and systematic ways. Pantomime is both an effective mechanism for communication when a common language is absent and an appropriate seedbed from which language could arise, mainly through cultural evolution. Taking inspiration from mimesis theory, we argued that the original uniquely human system of pantomime, including vocalization but dominated by gesture, is still present in modern humans and is put to use when we are denied the ability to use language. We looked at various contexts where language is blocked or constrained. Under such circumstances, people preferentially resort to pantomime, or elements of it, to bootstrap communication. In instances where these improvised communication systems (which we term ‘pantomimic fossils’) are used in a repeated interaction, they can develop language-like characteristics, possibly recapitulating the evolution of communication in our phylogeny. In our view, these facts explain why bodily mimesis and pantomime are not just relics of our evolutionary past. They have remained with us because they are extremely powerful tools for facilitating communication in the absence of a shared language.

## Footnotes

1 *Agent First* and *Topic Last* are semantic/pragmatic principles of ordering strings of signs. The principle *Agent Firs*t says the Agent should precede exponents of other semantic roles in the string. According to this principle, the string ‘tree hit Fred' would mean that the tree hit Fred and not that Fred hit the tree [2, p 247]. The principle *Topic Last* says that the informationally new element should be placed last in the string; e.g. in ‘In the room sat a bear', the noun phrase ‘a bear' is placed last for focal effect [2, p 248].

2 Understood broadly as information that identifies entities in the physical or phenomenological domain and asserts some relations between them [19, pp. 141–142].

3 According to mimesis theory, bodily mimesis laid the foundation not only for the emergence of language but also for other semiotic systems in modern human communication, such as co-speech gesture, depiction and music [13,23].

4 In this regard, iconic gesture differs from ape communication, which consists of a limited number of signals related to specific semantic domains [4,28].

5 As already noted §2b(i), it is difficult to find pure icons. Even the majority of primary iconic signs are not free elements of indexicality and conventionality. In our example, we should expect that the manner in which the hammering enactment is performed will, for example, reflect the praxic conventions of hammering in a particular community (e.g. related to the type of hammer that is commonly used, the type of objects that are commonly hammered, etc.).

6 Vocal iconic signs, e.g. for expressing emotional valence [39], supported gesture in the transmission of referential-propositional meaning. As argued by Levinson [41,42], vocalization may also have performed a range of non-significative functions related to coordinating interaction.

7 Expressing meaning through body movement (*Oxford English Dictionary*, https://www.oed.com).

8 In its most literal use, pantomime is able to communicate agentive actions performed by humans and, metonymically, human agents performing these actions [39]. However, by means of personification [45,46], pantomime is also able to refer to the behaviour of animals with the same body plan [47] as humans have (cf. the vertebrate body plan; [48]), and even in a more extended way refer to other animals, plants or inanimate objects by mapping the human body plan onto their structural characteristics (see example of the original sign for ‘airplane’ in STPSL, §3d).

9 For example, in the pantomime of hammering, there is a mapping of the whole body, although it is only the hand performing the hammering that is thematic. Zlatev and Andrén [58] contrast 1-person perspective with 3-person perspective, where the articulating parts of the body figure as observed objects, isolated from the rest of the body (which does not bear any relation to the represented object). The distinction into 1-person and 3-person perspective is similar to McNeill's [43] contrast between character- and observer-viewpoint but is more precise and less terminologically loaded (for a discussion, see [4]).

10 In tracing, the gesture follows the path of a moving object; in embodying, the hand or hands are used to stand for an object as a whole [56].

11 This does not mean that post-mimetic communication, language included, is bereft of iconicity. In fact, iconicity permeates different domains of language (both spoken and signed): phonology, semantics and syntax. For a review, see e.g. [61].

12 It is mainly described as ‘signe’, which in late Middle English and early Modern English typically referred to ‘gesture’ (*Oxford English Dictionary*, https://www.oed.com).

13 In neuropathology, the term ‘pantomime’ is commonly used, but is roughly equivalent to iconic gesture, often whole-body iconic gesture, and is a standard diagnostic tool for apraxia [72–74]. Sometimes, however, it is defined more narrowly as the imitation of a simple action, i.e. execution of the relevant motor sequence in the absence of its instrumental goal (the tradition going back to Hughlings Jackson [75]). For review, see [40].

14 The languages discussed here are in different stages of emergence, which affects the granularity with which we can look at the bootstrapping stage. In the older languages, ABSL and NSL, the bootstrapping stage refers to the communication of the first signers—from stratum I in the case of ABSL [103] and the first cohort in the case of NSL [104]. STPL has a much shorter history, which started in 2013, when an educational facility for deaf children was established; here, the bootstrapping stage refers to the first signs produced by the first children brought into this facility [92].

15 In personification, the structure of an object or an animal is mapped onto the human body, as in the example above, where the wings are mapped onto the hands ([45, p 4]; [46]).

16 Importantly, ‘pantomimic fossils’ would refer to these first signs, and—emphatically—not to the later stages of the developing system, which rapidly becomes fully linguistic and therefore not pantomimic.

17 ABSL started with four deaf siblings who were born in the 1930s (stratum I). The only available data on the earliest form of ABSL is video footage of a narrative produced by one of these original signers, who was then in his 60s [103].

18 The most recent reports of individual instances of pantomime or iconic gesture in great apes appear to strengthen rather than undermine this point. For example, Douglas and Moscovice [164] report instances of what they call a ‘form of pantomime’, but which is limited to a specific sexual context. Through an extensive analysis of published reports and data mining, Russon [165] was able to identify a total of 62 instances of great ape pantomime, most of them ‘simple, imperative, and scaffolded by partners' relationship and scripts’. This close-endedness is important. It shows that even if non-human apes can occasionally produce signs interpreted by human observers as pantomimic, they do not have pantomime as a system of communication—they lack the ability or motivation to systematically use their body as a representational device.

## Dictionaries

Oxford English Dictionary, www.oed.com

## Data accessibility

This article has no additional data.
